# Supplementary material for: The causal relationship between the human gut microbiota and pyogenic arthritis: a Mendelian randomization study
Source: Front Cell Infect Microbiol. 2024 Nov 26;14:1452480. doi: 10.3389/fcimb.2024.1452480 (PMC11629706; doi:10.3389/fcimb.2024.1452480)
Supplement: Supplementary file 1 [file DataSheet1.zip › mendelian test/ebi-a-GCST90027740.csv_leave_one_out.pdf]

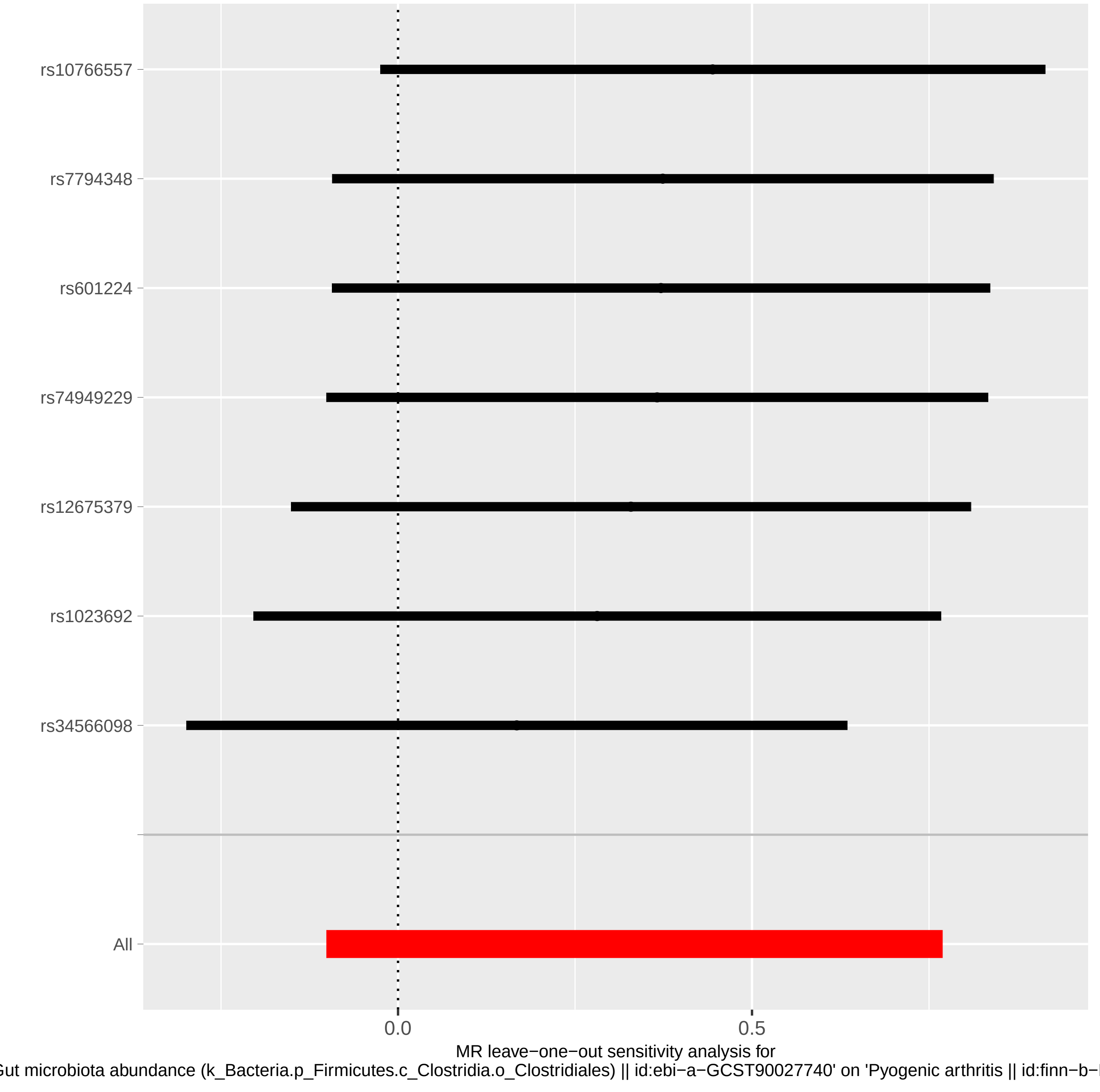
rs10766557
rs7794348
rs601224
rs74949229
rs12675379
rs1023692
rs34566098
All
0.0
0.5
MR leave−one−out sensitivity analysis for
Gut microbiota abundance (k_Bacteria.p_Firmicutes.c_Clostridia.o_Clostridiales) || id:ebi−a−GCST90027740' on 'Pyogenic arthritis || id:finn−b−
